# Olive Leaf Polyphenols (OLPs) Stimulate GLUT4 Expression and Translocation in the Skeletal Muscle of Diabetic Rats

**DOI:** 10.3390/ijms21238981

**Published:** 2020-11-26

**Authors:** Jasminka Giacometti, Damir Muhvić, Tanja Grubić-Kezele, Marina Nikolić, Tamara Šoić-Vranić, Snježana Bajek

**Affiliations:** 1 Department of Biotechnology, University of Rijeka, Radmile Matejčić 2, 51000 Rijeka, Croatia; 2 Department of Physiology and Immunology, Faculty of Medicine, University of Rijeka, Braće Branchetta 20, 51000 Rijeka, Croatia; damir.muhvic@medri.uniri.hr (D.M.); tanja.grubic@medri.uniri.hr (T.G.-K.); 3 Clinical Department for Clinical Microbiology, Clinical Hospital Center Rijeka, Krešimirova 42, 51000 Rijeka, Croatia; 4 Department of Anatomy, Faculty of Medicine, University of Rijeka, Braće Branchetta 20, 51000 Rijeka, Croatia; marina.nikolic@medri.uniri.hr (M.N.); tamara.soic.vranic@medri.uniri.hr (T.Š.-V.); snjezana.bajek@medri.uniri.hr (S.B.)

**Keywords:** skeletal muscle, diabetes mellitus, glucose transporter 4, GLUT4 translocation, Rab8A, Rab13, Rab14

## Abstract

Skeletal muscles are high-insulin tissues responsible for disposing of glucose via the highly regulated process of facilitated glucose transporter 4 (GLUT4). Impaired insulin action in diabetes, as well as disorders of GLUT4 vesicle trafficking in the muscle, are involved in defects in insulin-stimulated GLUT4 translocation. Since the Rab GTPases are the main regulators of vesicular membrane transport in exo- and endo-cytosis, in the present work, we studied the effect of olive leaf polyphenols (OLPs) on Rab8A, Rab13, and Rab14 proteins of the rat soleus muscle in a model of streptozotocin (SZT)-induced diabetes (DM) in a dose-dependent manner. Glucose, cholesterol, and triglyceride levels were determined in the blood, morphological changes of the muscle tissue were captured by hematoxylin and eosin histological staining, and expression of GLUT4, Rab8A, Rab13, and Rab14 proteins were analyzed in the rat soleus muscle by the immunofluorescence staining and immunoblotting. OLPs significantly reduced blood glucose level in all treated groups. Furthermore, significantly reduced blood triglycerides were found in the groups with the lowest and highest OLPs treatment. The dynamics of activation of Rab8A, Rab13, and Rab14 was OLPs dose-dependent and more effective at higher OLP doses. Thus, these results indicate a beneficial role of phenolic compounds from the olive leaf in the regulation of glucose homeostasis in the skeletal muscle.

## 1. Introduction

Glucose metabolism is a good example of communication and signalling in the organism, and the investigation of its transport and delivery in specific tissues such as skeletal muscle is important for the maintenance of the whole-body glucose homeostasis [1]. The glucose transporter 4 (GLUT4) controls cellular glucose transport into skeletal muscles and adipose tissues in response to insulin stimulation [2]. In the basal state, GLUT4 is sequestered in intracellular storage vesicles (GSV) in the cytoplasm. After insulin stimulation, GLUT4 translocation is accelerated to the plasma membrane (PM) via two complex systems: signal transduction and vesicular transport [3]. Two pathways have been identified in the mechanism of GLUT4 translocation stimulated by insulin, (i) the protein kinases-activated pathways including phosphoinositide-3-kinase (PI3K) and the mitogen-activated protein kinase 3/1 (MAPK3/1, ERK1/2), and (ii) the Cb1-CAP-CrkII-C3G-TC10 pathway. The regulation of GLUT4 trafficking proteins is a complex process, which includes small GTPases, tethering complexes and the vesicle fusion machinery in transmitting the effects of insulin [4]. Likewise, the correct functioning of the PI3K/AKT, MAPK, and AMPK pathways is essential for proper metabolic control. Thus, their dysfunction impairs glucose homeostasis [5,6].

Rab-GTPases, as molecular switchers, are central regulators of vesicular transport along exocytic, endocytic, and recycling pathways [3]. Altered expression levels of Rab molecules in these pathways, including both activation and inactivation, have been reported in various diseases [7]. Based on their tissue-specific role and specific mechanism in the regulation of intracellular GLUT4 trafficking, we focused on the Rab8A, Rab13, and Rab14 in the soleus muscle. In the steady-state, Rab8A can be found in the membrane of secretory vesicles in the exocytic pathway to plasma membrane (PM), Rab14 in early endosome for endosomal sorting of GLUT4 cargos, while Rab13 in recycling endosome for endosomal sorting and PM remodeling [7]. However, these associated Rabs do not necessarily co-localize on the same vesicles.

Since the skeletal muscles are one of the primary sites for the dietary glucose disposal, their function depends on the insulin-stimulated mobilization of GLUT4 vesicles to its cell surface. In rodent skeletal muscle, insulin phosphorylates AS160, and after its mutation, impairs insulin-stimulated skeletal muscle glucose uptake [8]. Moreover, site-specific phosphorylation of the Rab-GTPase-activating proteins AS160 and TBC1D1 are critical for GLUT4 translocation of glucose uptake [9]. Recently, it has been reported that the Rab-GAP AS160 controls the activity of the small GTPases Rab8A, Rab13, and Rab14 in muscle cells [10,11] and Rab10 and Rab14 in adipocytes [12,13,14,15], needed for GLUT4 vesicle mobilization [4,16]. Although insulin induces activation of the Rab8A and Rab13 in the GLUT4 translocation, overexpression of Rab8A can partially disable blocking of insulin-stimulated GLUT4 translocation exerted by AS160-4A in muscle cells. Rab8A and perhaps Rab14 are targets of the insulin-regulated Rab-GAP AS160 in the regulation of GLUT4 traffic in muscle cells [11]. In a model, whereby AS160, Rab8A, and myosin Vb (MyoVb) are part of a linear signaling cascade required for insulin-induced GLUT4 translocation in muscle cells, overexpression of a fragment of MyoVb can inhibit insulin-induced GLUT4 translocation and modify the subcellular distribution of GTP-loaded Rab8A [17]. Rab8A, besides being involved in glucose metabolism, may be involved in regulating lipid metabolism and may have an important metabolic role in skeletal muscle. Thus, downregulation of Rab8A inhibits the insulin-stimulated translocation of GLUT4 [18,19,20]. Additionally, Rab8A plays an important role in lipid droplets fusion mediated by fat-specific protein 27 (FSP27) that regulates the storage of triacylglycerol and Rab8A interacting protein, MSS4, in 3T3-L1 adipocytes [19]. Based on what was previously mentioned, we suppose that overexpression of Rab8A could have a positive effect on the reduced formation of lipid droplets and their accumulation in skeletal muscles, especially in oxidative ones [20].

**Figure A1:**
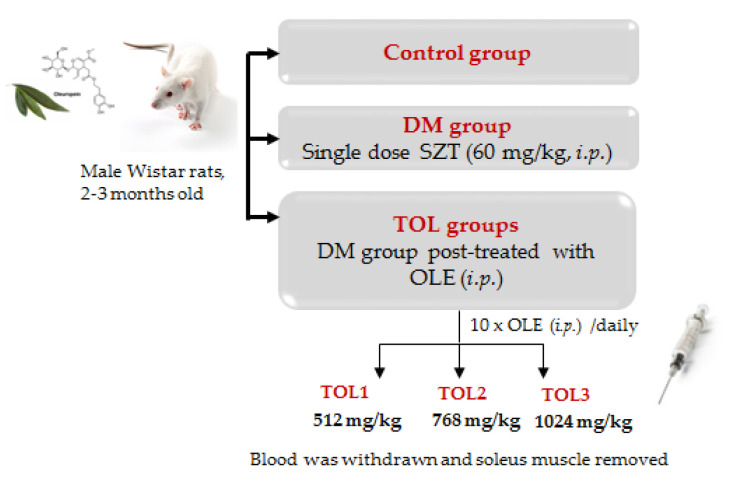
Experimental design.

The use of bioactive phenolic compounds is recognized as one of the strategies for the treatment and prevention of diabetes [21]. Due to diminished translocation and trafficking of GLUT4 vesicles in skeletal muscles in diabetes [18,22], our investigation in vivo is directed toward new insights in roles of specific Rab molecules in diabetes as the possible targets for therapy with polyphenols and potential improvement of the diabetic state after the treatment. Namely, the mechanism by which insulin regulates the intake, delivery, and transport of glucose to the tissues under the influence of bioactive phenolic compounds has not yet been clarified. That especially relates to intracellular vesicular transport of the GLUT4. On the other hand, numerous in vitro and in vivo studies are based on signal transduction via protein kinase-activated pathways, demonstrating the effect of plant polyphenols on the GLUT4 translocation [23], as presented in Appendix A Figure A1 Table A1. However, a limited number of studies refer to the effects of polyphenols on Rab GTPases in the regulation of GLUT4 trafficking. Samad et al. [24] reported that [6] -Gingerol increased glucose uptake by increasing the membrane docking of GLUT4 in skeletal muscle and increasing Rab8 and Rab10 in the treatment of a Lepr^db/db^-type diabetic mouse model. Likewise, resveratrol promoted Rab7 expression in vivo and in vitro by improving impaired autophagic function in cardiac muscles in diabetic cardiomyopathy, thus influencing the translocation of GLUT4 through the late endosomal pathway [25]. Based on the fact that autophagy and GLUT4 trafficking can be overlapping in the mechanism of endocytosis and intracellular compartments, using similar proteins for exocytosis, as well as that PI3K and AMPK also control autophagy and GLUT4, control of GLUT4 autophagy trafficking could enable a new direction in the study of diabetes in the future [26].

Oleuropein (OL), as the most abundant phenolic compound in olive leaf extract, significantly enhanced glucose uptake and the phosphorylation of AMPK and MAPKs, but not PI3K in PI3K/Akt in C2C12 muscle cells [27,28]. However, there is no evidence about the effect of oleuropein on intracellular vesicular GLUT4 trafficking. Given that oleuropein increases GLUT expression through the improving signal transduction of protein kinases-activated pathways in muscle cells, this study was conducted to observe the effect of olive leaf polyphenols on impaired intracellular vesicular glucose transport in diabetes. We hypothesized that diabetes inhibits specific Rab GTPases in GLUT4 trafficking and that olive leaf polyphenols reactivate them. To test this hypothesis, we investigated the effect of olive leaf polyphenols (OLP) on Rab8A, Rab13, and Rab14, as well as GLUT4 in the soleus muscle of rats with streptozotocin-induced diabetes (SZT). Therefore, the main objective of this study was to investigate the translocation of GLUT4 in skeletal muscle by stimulating specific Rab GTPases by oleuropein-rich olive leaf extract in maintaining glycemic control. Our results provide evidence for the role of Rab8A, Rab13, and Rab14 in the regulation of GLUT4 translocation and controlling glucose metabolism in the skeletal muscle.

## 2. Results

### 2.1. Glucose Tolerance Test (GTT)

**Figure 1:**
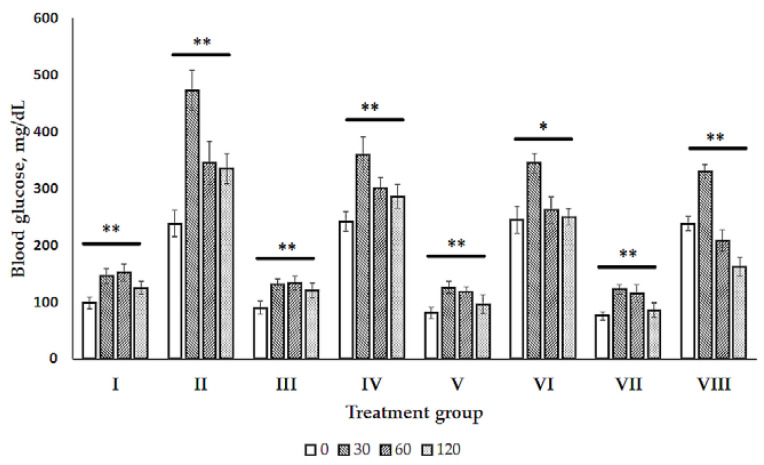
Glucose tolerance test (GTT) of normal (healthy) and diabetic rats. The data shown are of the group **I**—normal control, group **II**—diabetic control, group **III**—normal control administrated by 512 mg/kg olive leaf extract (OLE), group **IV**—diabetic control administrated by 512 mg/kg olive leaf extract (OLE), group **V**—normal control administrated by 768 mg/kg olive leaf extract (OLE), group **VI**—diabetic control administrated by 768 mg/kg olive leaf extract (OLE), group **VII**—normal control administrated by 1024 mg/kg olive leaf extract (OLE), group **VIII**—diabetic control administrated by 1024 mg/kg olive leaf extract (OLE). Blood glucose level was monitored at the start (0 min) and 30, 60, and 120 min after the OLE administration. Differences between the normal and diabetic rats at the time 0, 30, 60, and 120 min of treatment were determined by nonparametric Kruskal–Wallis analysis of variance (ANOVA) by Ranks test. The difference was significant at * *p* < 0.05 and ** *p* < 0.01.

The glucose tolerance test (GTT) was carried out to evaluate the effect of different doses of olive leaf extract (512, 768, and 1024 mg/kg body weight, BW) on glucose tolerance in normal rats and in rats with the previous administration of SZT (see Figure 1). After 30, 60, and 120 min of the glucose intraperitoneal administration (*i.p.*), the blood glucose level in diabetic rats was higher than in normal rats.

A time course of the blood glucose level differed and changed in the normal control group compared with the diabetic group as well as in the normal and diabetic groups with OLP therapy. The statistical significant interactions between glucose and independent variables (group, time) in the multivariate generalized linear model (GLM) was reached using analysis of variance (ANOVA) or multivariate analysis of variance (MANOVA) main effects. ANOVA/MANOVA main effects confirmed the findings: F_Group×Time_ = 42.86, *p* < 0.0001 for healthy and diabetic control, for healthy and diabetic TOL1 group (F_Group×Time_ = 21.60, *p* < 0.0001), for healthy and diabetic TOL2 group (F_Group×Time_ = 23.84, *p* < 0.0001), and for healthy and diabetic TOL3 group (F_Group×Time_ = 75.61, *p* < 0.0001).

Although ending blood glucose levels did not change significantly in comparison to the starting ones, the significant changes in the blood glucose level were obtained at the time course points 30 and 60 for healthy control rats (*p* = 0.0106 and *p* = 0.0055, respectively), TOL1 rats (*p* = 0.0197 and *p* = 0.0073, respectively), and TOL3 rats (*p* = 0.0050 and *p* = 0.0300, respectively), while blood glucose in the TOL2 rats changed significantly at 30 min (*p* = 0.0031). Taken together, the obtained results showed the hypoglycemic effect of OLP in diabetic rats in a dose-dependent manner. The dose of olive leaf extract 1024 mg/kg (44.5 mg/kg oleuropein) indicates the most beneficial effect due to the decreased starting glucose level in diabetic rats, as shown in Figure 1. However, that should be confirmed further over a longer treatment period than that 120 min.

### 2.2. Blood Biochemistry

Animals that remained alive during experiments were taken into account in this study. As shown in Table 1, significant changes among all studied groups were found in animal weight (*p* = 0.005), blood glucose (*p* = 0.000), and blood triglycerides (*p* = 0.005). After SZT-induced diabetes, the blood glucose level was significantly higher than those in the healthy control group, while after 10 days of treatment with olive leaf extract (OLE), the blood glucose level was significantly lower than those in the untreated diabetic group, as expected. Diabetic animals showed significant loss of body weight. In addition, animals treated with OLE in dose-dependent groups TOL1, TOL2, and TOL3 also showed a loss of body weight, but not significantly.

Treatment with OLE significantly reduced level of blood triglycerides in the TOL1 and TOL3 groups compared to the diabetic control group as well as increased in the untreated diabetic group compared to healthy control. These results indicated that OLE, in a dose-dependent manner, has the potential to improve hypertriglyceridemia to SZT-induced diabetic rats.

### 2.3. Morphological Analysis

**Figure 2:**
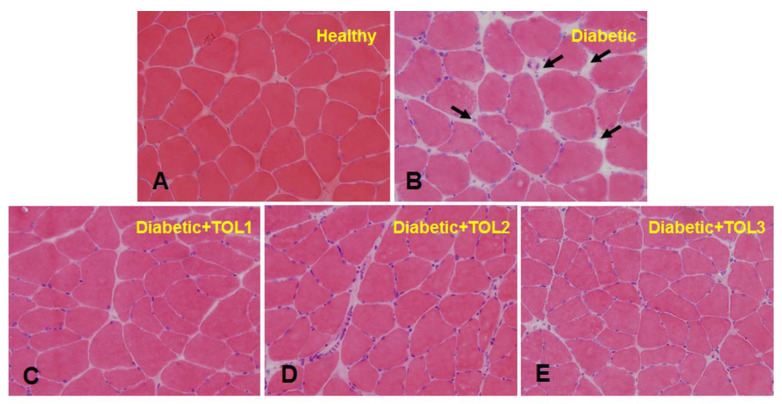
Representative histological evaluation of hematoxylin and eosin-stained soleus cross-sections in (**A**)—healthy control, (**B**)—diabetic control, (**C**–**E**)—the post-treated diabetic state with a dose-dependent OLP (TOL1, TOL2, TOL3, respectively). Black arrows sign increased endomysium in the diabetic control group (**B**). Magnification ×40.TOL1-diabetic group treated with 512 mg/kg olive leaf extract (OLE), TOL2-diabetic group treated with 768 mg/kg olive leaf extract (OLE), and TOL3-diabetic group treated with 1024 mg/kg olive leaf extract (OLE).

Diabetic muscle is accompanied by morphological change. To get insights into the possible structural changes, histological analyses were performed by hematoxylin and eosin (H&E) staining in the planar muscle, i.e., the cross-sectional area of the soleus sections (Figure 2).

The Control group (Figure 2A) demonstrated normal fibers with a preserved polygonal shape, peripheral myonuclei in a subsarcolemmal position near the cell membrane, and preserved fascicular pattern, with no apparent changes in the endomysium, except for the presence of blood capillaries. The sarcoplasm seems relatively uniform throughout the cell.

Histopathological examination of the diabetic muscle (Figure 2B) revealed hypertrophic processes, loss of polygonal shape, and decrease in the fiber size and enlarged peripheral nuclei. Also discerned have been muscle fibers with reduced area and atrophic fibers along with thicker connective tissue. As presented, the cross-section area in the rat soleus was characterized by an increased endomysium in diabetic rats in relation to the healthy control (Figure 2A,B, indicated by black arrows). In addition, the diabetic groups treated with OLE (Figure 2C–E) showed an increase in peripherally placed nuclei with changes in the perimysium and increased area of muscle fibers. These changes were found in the TOL2 and the TOL3 groups, where fibers were polygonal-shaped and tissue organization was normal. Results show that dose-dependent OLE treatment was able to improve the muscle cytoarchitecture of diabetic rats and could maintain the mass or strength of intramuscular connective tissues by re-establishing the regulation of muscle proteins.

### 2.4. GLUT4 Expression on Muscle Fiber Membrane

**Figure 3:**
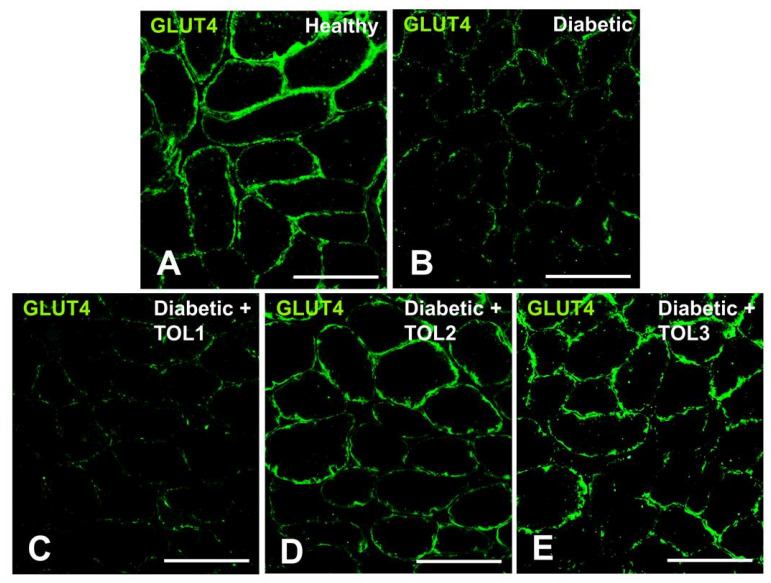
Representative GLUT4 immunofluorescence images in cross-sections of muscle fibers in rat soleus. Images (**A**–**E**) show GLUT4 localization in green (magnification 400×). Bars indicate 100 µm.

The plasma membrane provides a high surface area for insulin delivery to the muscle cell and glucose uptake observed as cross-striations in the immunofluorescence imaging of muscle fibers (Figure 3A–E). In the rat soleus in the control group, GLUT4 immunofluorescence staining appears as a cytosolic vesicle found in the intracellular layers and small punctuate spots found in the perinuclear region. Since cytosolic vesicles were less frequent, GLUT4 was observed more in the PM. The accumulation of GLUT4 in the PM of the healthy muscles is highly uniform in comparison to those seen in diabetic muscles (Figure 3A–E), with less accumulation of GLUT4 in the PM, as well as the absence of vesicles in intracellular layers.

After OLE therapy, integrity of translocated GLUT4 on the membrane of muscle fibers was observed (Figure 3D,E). The highest effect of OLE treatment on GLUT4 translocation was found in the groups TOL2 (Figure 3D) and TOL3 (Figure 3E). Considering the obtained results, we could suggest that olive leaf polyphenols affect the regulatory mechanisms controlling GLUT4 translocation to the membrane surface in a dose-dependent manner. However, the mechanisms of intracellular GLUT4 translocation are dependent on the consumption of bioactive phenolic and possible targets are still unknown.

### 2.5. GLUT4/Rab8A, GLUT4/Rab13, and GLUT4/Rab14 Colocalizations on Muscle Fiber Membrane

**Figure 4:**
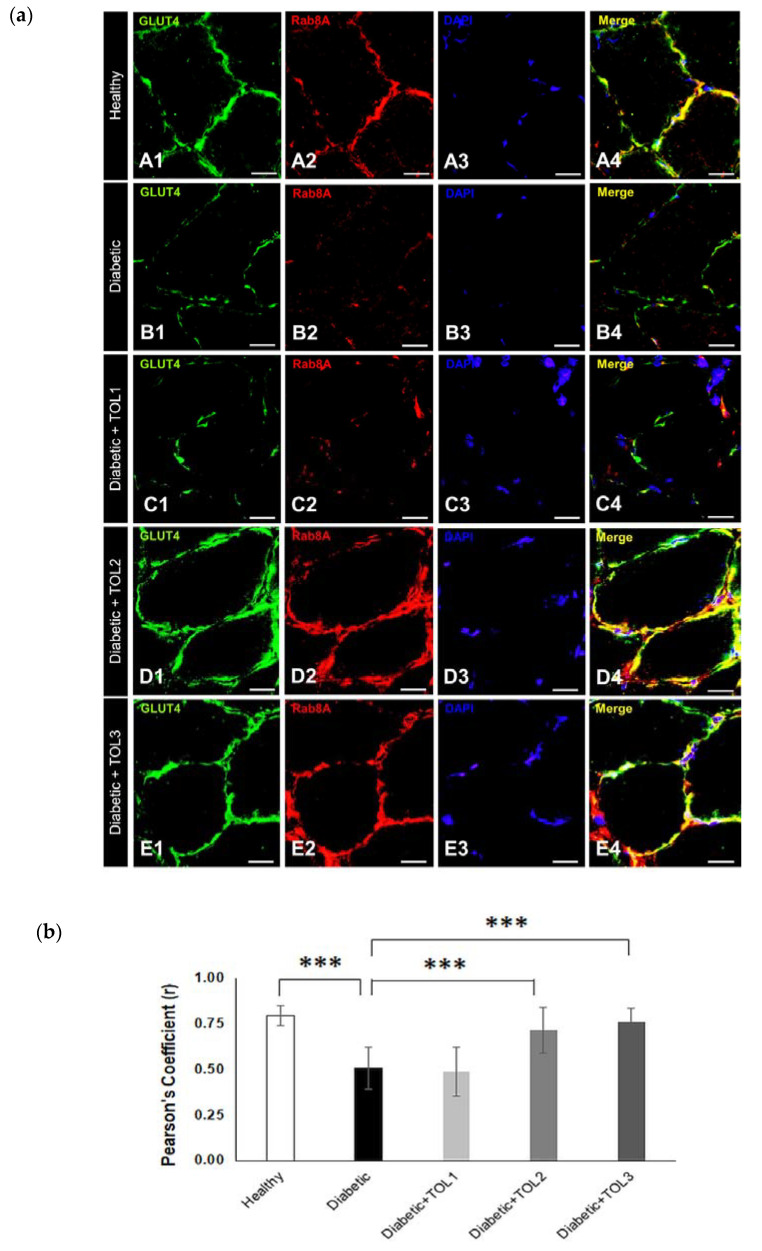
Colocalization of GLUT4 and Rab8A in cross-section muscle fibers of rat soleus. In the cross-section muscle fibers of rat soleus treated with the therapy of olive leaf (TOL) in a dose-dependent manner, fibers are present that abundantly express Rab8A. 4′,6-diamidino-2-phenylindole (DAPI) was used to stain nuclei (blue). Images (**a**(**A**–**E**)) show magnification 1000×. Scale bars indicate 20 μm. (**a**) Representative immunofluorescent pictures show the relationship between Rab8A and GLUT4 muscle fibers in Wistar rats: (**a**(**A1**–**A4**)) untreated; (**a**(**B1**–**B4**)) with SZT-induced diabetes; (**a**(**C1**–**C4**)) with SZT-induced diabetes and treated with TOL1, (**a**(**D1**–**D4**)) with TOL2, and (**a**(**E1**–**E4**)) with TOL3. (**b**) GLUT-Rab8A colocalization was assessed in the area of interest (0.014 mm^2^/4 μm slice × 3 slices/rat × 6 rats/group) by calculating the Pearson correlation coefficient using the Just Another Colocalization Plugin (JACoP plugin) on ImageJ. Values are expressed as mean ± standard deviation (SD). One-way ANOVA followed by the post hoc Scheffé test: *** *p* < 0.001.

**Figure 5:**
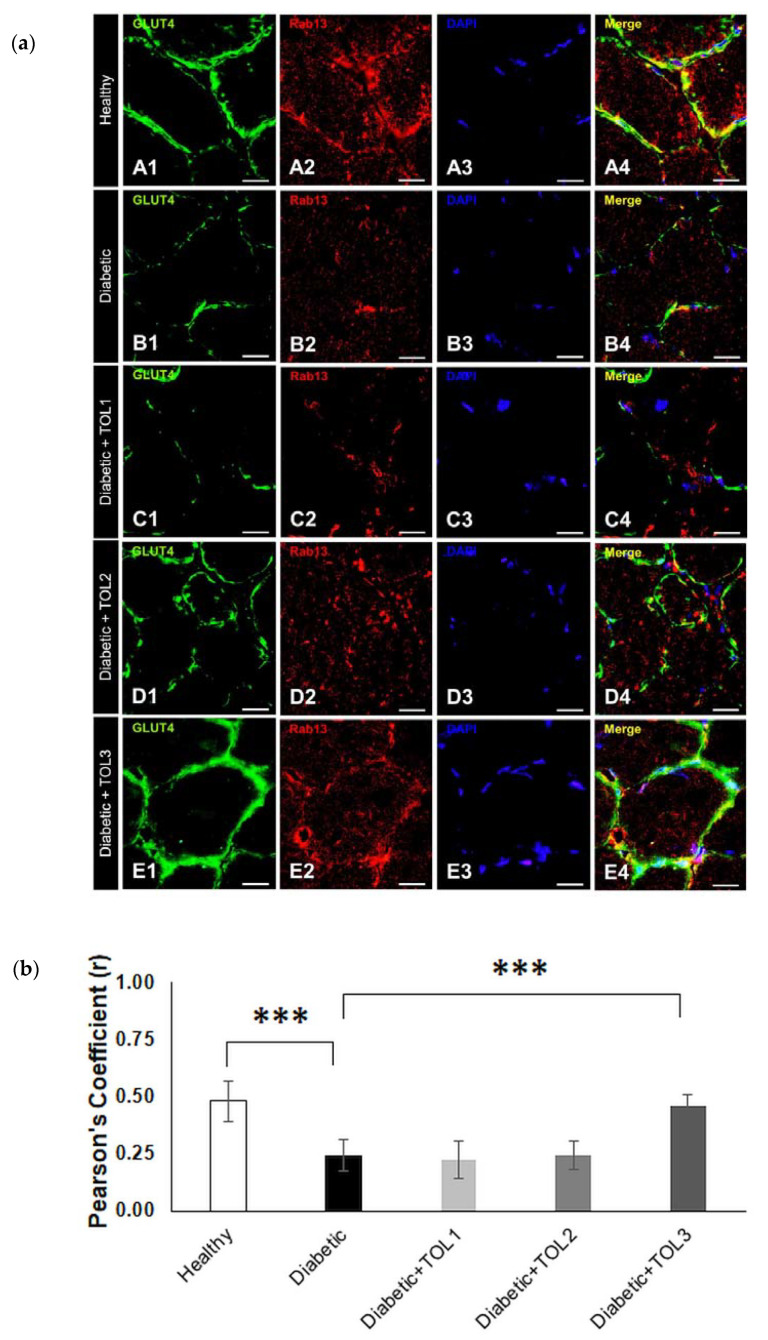
Colocalization of GLUT4 and Rab13 in cross-section muscle fibers of rat soleus. In the cross-section muscle fibers of rat soleus treated with the therapy of olive leaf (TOL) in a dose-dependent manner, fibers are present that express Rab13. DAPI was used to stain nuclei (blue). Images (**a**(**A**–**E**)) show magnification 1000×. Scale bars indicate 20 μm. (**a**) Representative immunofluorescent pictures show the relationship between Rab13 and GLUT4 muscle fibers in Wistar rats: (**a**(**A1**–**A4**)) untreated; (**a**(**B1**–**B4**)) with SZT-induced diabetes; (**a**(**C1**–**C4**)) with SZT-induced diabetes and treated with TOL1, (**a**(**D1**–**D4**)) with TOL2, and (**a**(**E1**–**E4**)) with TOL3. (**b**) GLUT-Rab13 colocalization was assessed in the area of interest (0.014 mm^2^/4 μm slice × 3 slices/rat × 6 rats/group) by calculating the Pearson correlation coefficient using the JACoP plugin on ImageJ. Values are expressed as mean ± SD. One-way ANOVA followed by the post hoc Scheffé test: *** *p* < 0.001.

**Figure 6:**
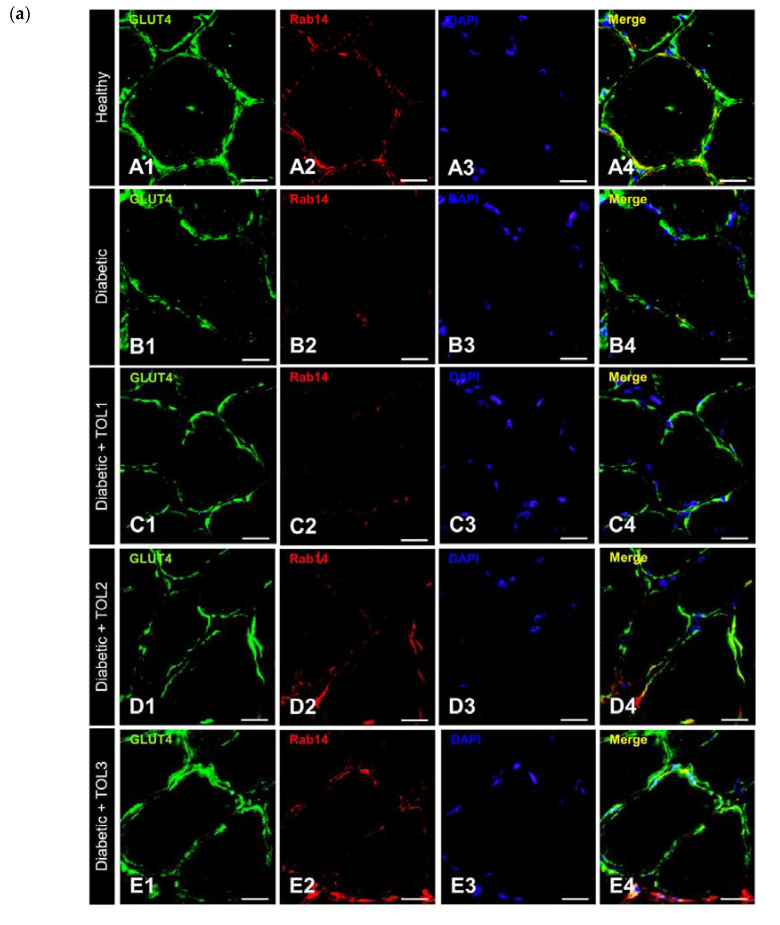

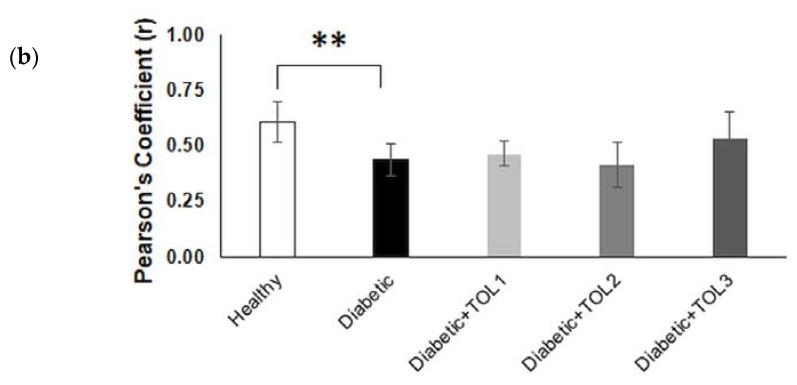
Colocalization of GLUT4 and Rab14 in cross-section muscle fibers of rat soleus. In the cross-section muscle fibers of rat soleus treated with the therapy of olive leaf (TOL) in a dose-dependent manner, fibers are present that express Rab14. DAPI was used to stain nuclei (blue). Images (**A**–**E**) show magnification 1000X. Scale bars indicate 20 μm. (**a**) Representative immunofluorescent pictures show the relationship between Rab14 and GLUT4 muscle fibers in Wistar rats: (**a**(**A1**–**A4**)) untreated; (**a**(**B1**–**B4**)) with SZT-induced diabetes; (**a**(**C1**–**C4**)) with SZT-induced diabetes and treated with TOL1, (**a**(**D1**–**D4**)) with TOL2, and (**a**(**E1**–**E4**)) with TOL3. (**b**) GLUT-Rab14 colocalization was assessed in the area of interest (0.014 mm^2^/4 μm slice × 3 slices/rat × 6 rats/group) by calculating the Pearson correlation coefficient using the JACoP plugin on ImageJ. Values are expressed as mean ± SD. One-way ANOVA followed by the post hoc Scheffé test: ** *p* < 0.01.

Having regard to the fact that polyphenols improve acute insulin secretion and insulin sensitivity, we hypothesize that they may participate in the activation of Rab8a and Rab13 and cause insulin-stimulated GLUT4 translocation in muscle. Rab8A expression and localization was evaluated in the tissue samples of the soleus, taken from the healthy, diabetic, and diabetic-treated animals. To visualize the interaction between GLUT4 and Rab8A after the used therapies, double immunofluorescence was used. The results of the colocalization analyses are reported as means and standard deviation (SD) (n = 6 from each experimental group for each colocalization analysis). The data were compared with findings obtained from the OLE, healthy control, and diabetic control animals. Representative immunofluorescence images of our data are presented in Figure 4, Figure 5 and Figure 6. Due to excessive fiber swelling in the diabetic muscle, the number of intact myofibers was reduced, thus leading to a decrease in GLUT4 expression in the observed muscle area (Figure 3B). A higher dose of OLE in the groups TOL2 and TOL3 induced a higher perinuclear deposition of GLUT4 (Figure 3D,E).

Rab8A was less localized in the intracellular matrix and was the most localized in the PM, in colocalization with GLUT4 (Figure 4a). The data clearly showed the highest GLUT4 and RabA8 expression and colocalizations at the muscle fiber membrane in the groups TOL2 (Figure 4a(D1–D4),b, Pearson’s Coefficient = 0.761) and TOL3 (Figure 4a(E1–E4),b, Pearson’s Coefficient = 0.714). On the other hand, the lowest administered dose of OLE had a less effect on Rab8A expression (Figure 4a(C1–C4),b, Pearson’s Coefficient = 0.488). In relation to healthy control, a significant lower GLUT4/Rab8A colocalization (*p* = 0.000001) was found in diabetic muscle. Additionally, a significant higher GLUT4/Rab8A colocalization was observed in TOL2 and TOL3 groups (*p* = 0.000575 and *p* = 0.000016, respectively) in relation to the diabetic control group, as shown in Figure 4b.

Next, OLE therapies have a similar effect on Rab13 expression (Figure 5a). The highest GLUT4 and Rab13 colocalizations are found at the muscle fiber membrane in the groups TOL2 (Figure 5a(D1–D4),b, Pearson’s Coefficient = 0.243) and TOL3 (Figure 5a(E1–E4),b, Pearson’s Coefficient = 0.458). The OLE therapy with the lowest dose had a minimum effect on Rab13 expression (Figure 5a(C1–C4),b, Pearson’s Coefficient = 0.224). In addition, Rab13 was found abundantly in intracellular space in healthy and diabetic TOL2 and TOL3 groups, and we hypothesize that OLE promotes the recruitment of Rab8A and Rab13 and stimulates them to participate in insulin-stimulated GLUT4 translocation. A significant lower GLUT4/Rab13 colocalization (*p* = 0.000000) was found in diabetic muscle than those in the healthy control group. However, in relation to the diabetic group, a significant higher colocalization was observed only in the TOL3 group (*p* = 0.000000), as shown in Figure 5b. That suggests that activation of Rab13 and colocalization with GLUT4 is OLE dose-dependent. As reported by Sun et al. [10], Rab8A activation peaked earlier than Rab13 activation, thus insulin signaling may successively activate Rab8A and Rab13 and modulate the regulation of GLUT4 translocation by these two Rab proteins in muscle cells. However, we focused here on the accumulation of Rab13 near PM, showing their involvement in regulating the transport of GLUT4 to PM.

To evaluate the role of Rab14 in GLUT4 translocation to PM under the diabetic state and OLE treatment in vivo, double immunofluorescence staining was performed in cross-section muscle fibers. Significantly reduced GLUT4/Rab14 colocalization was found in diabetic rats (*p* = 0.001261) (Figure 6a(A1–A4),(B1–B4)).

As expected, the highest GLUT4 and Rab14 colocalization are found at the muscle fiber membrane in the healthy group (Figure 6a(A1–A4),b, Pearson’s Coefficient = 0.610) compared to the diabetic group (Figure 6a(B1–B4),b, Pearson’s Coefficient = 0.441). In OLE-treated diabetic groups (TOL1, TOL2, TOL3, shown in Figure 6a(C1–C4),(D1–D4),(E1–E4)),b, with Pearson’s Coefficient 0.468, 0.416, 0.536, respectively) did not observe a significant change. That suggested that Rab8A and Rab13 are independent of Rab14.

### 2.6. GLUT4, Rab8A, Rab13, and Rab14 Expression in Rat Soleus

**Figure 7:**
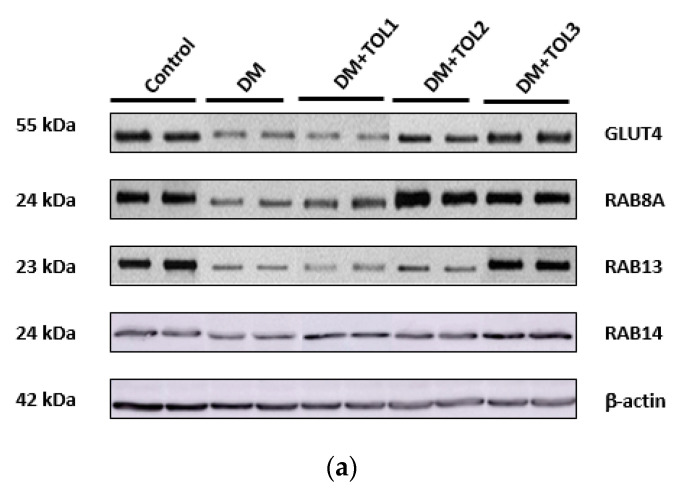

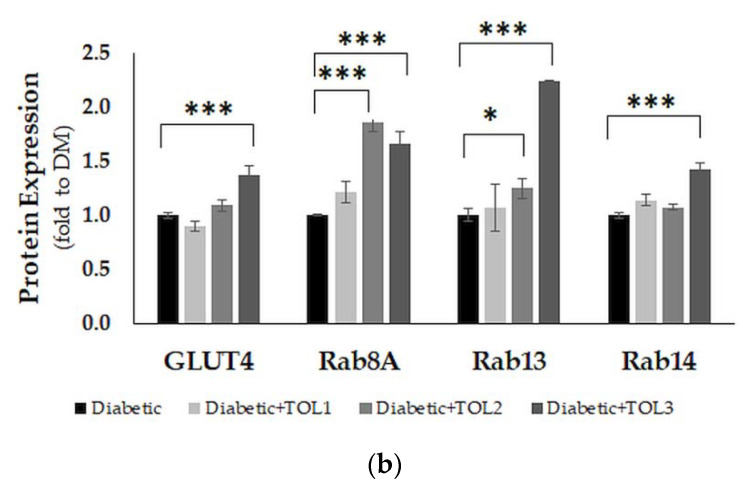
Immunoblot of GLUT4, Rab8A, Rab13, and Rab14 in the isolated rat soleus muscle proteins. Cell lysate proteins (50 µg) were immunoblotted using β-actin as the loading control. (**a**) Representative Western blot images of the target proteins. (**b**) The expression of GLUT4, Rab8A, Rab13, and Rab14 are shown at the normalized expression level of DM. For each group, values are presented as the mean ± SD of six rats per group. One-way ANOVA followed by the post hoc Scheffé test were used for the statistical analysis: * *p* < 0.05 and *** *p* < 0.001.

The expression of GLUT4, Rab8A, Rab13, and Rab14 was analyzed by Western blot (WB) analysis in the rat soleus muscle (Figure 7a). GLUT4 (*p* = 0.010), Rab8A (*p* = 0.012), Rab13 (*p* = 0.010), and Rab14 (*p* = 0.022) were changed significantly among all examined groups. WB revealed significant suppression of GLUT4 (*p* = 0.000), Rab8A (*p* = 0.000), and Rab13 (*p* = 0.000) in the diabetic group compared to the healthy control (Figure 7b), while Rab14 decreased in the diabetic group, but not significantly. OLE therapies significantly changed expression of GLUT4, Rab8A, Rab13, and Rab14 in diabetic rats: in the TOL1 group decreased GLUT4 (*p* = 0.011), in the TOL2 group increased GLUT4 (*p* = 0.017), Rab8A (*p* = 0.000), and Rab13 (*p* = 0.002), and in the TOL3 group increased GLUT4 (*p* = 0.000), Rab8A (*p* = 0.000), Rab13 (*p* = 0.000), and Rab14 (*p* = 0.000).

This means that OLE therapy improved expression of GLUT4 by 1.37-fold in the TOL3, Rab8A by 1.86-fold, and 1.66-fold in the TOL2 and TOL3 groups respectively, Rab13 by 2.25-fold in the TOL3, and Rab14 by 1.43-fold in the TOL3 group, compared with the diabetic group.

Further, we found a significant correlation between GLUT4 and Rab8A (r = 0.615), Rab13 (r = 0.916), and Rab14 (r = 0.685) expression levels, as well as between Rab8a and Rab13 (r = 0.592) for all examined groups. Likewise, by using multiple regression analysis, a significant correlation between GLUT4 expression level and Rab8a (*p* = 0.015), Rab13 (*p* = 0.000), and Rab14 (*p* = 0.005) was found, as well as between Rab8A and Rab13 (*p* = 0.020). However, the correlation between Rab8A and Rab14 was not established. Since there existed differences between Rab8A and Rab13 in the TOL2 and TOL3 groups, we supposed that the steps in GLUT4 translocation were dependent on OLP dose. The reason could also be in the re-establishment of cellular architecture after possible damage caused by diabetes dependent on OLP dose, which is crucial for the establishment of the conditions for GLUT4 exocytosis and, consequently, tethering of the GLUT4 vesicle to the PM. These findings suggested that translocation of GLUT4 in skeletal muscles is insulin-dependent and that it can be stimulated and modified with OLP in a dose-dependent manner.

All three examined Rab-GTPases regulated GLUT4 trafficking. The interaction between Rab8A and Rab13 was confirmed, while Rab14 was independent of the previous two. In addition, OLP activated Rab8A, Rab13, and Rab14, and thus stimulated GLUT4 translocation in the soleus muscle in diabetic rats in a dose-dependent manner. Furthermore, it will be interesting to investigate the effect of OLP on glucose metabolism, transport, and delivery in other skeletal muscles (e.g., tibialis, gastrocnemius), depending on their functionality and ability for the maintenance of glucose uptake.

## 3. Discussion

Various authors reported that diabetes decreases the expression and translocation of GLUT4 at the muscle membrane and how phenolic compounds may stimulate GLUT4 translocation and thus improve glucose uptake [29,30,31,32,33,34]. However, the mechanisms by which polyphenols contribute to the improvement of GLUT4 translocation and glucose uptake are still unknown, especially at the level of GLUT4 vesicle trafficking. In addition, numerous studies reported that dietary polyphenols modulate carbohydrate and lipid metabolism, and diminish hyperglycemia, dyslipidemia, and insulin resistance [35,36,37,38,39,40]. Thus, this work aimed to investigate insulin-stimulated translocation of GLUT4 through activation of specific Rab GTPases in intracellular vesicular transport of GLUT4 in skeletal muscle using the therapy by olive leaf polyphenols in diabetic rats in a dose-dependent manner.

### 3.1. Olive Leaf Polyphenols Reduced Hyperglycemia and Hyperlipidemia

Here, we found that OLP treatments (marked as TOL1, TOL2, and TOL3) significantly reduced the blood glucose level of diabetic rats compared to those untreated (DM) (see Table 1). Previously, the GGT was carried out to evaluate the effect of different doses of olive leaf extract in healthy and diabetic rats, and the dose of olive leaf extract 1024 mg/kg (44.5 mg/kg oleuropein) was indicated as the most beneficial (see Figure 1). In addition, OLE administration provoked reducing blood triglycerides in the TOL1 and TOL3 groups compared to the DM group. These beneficial effects can be attributed to oleuropein (OL) and their metabolites tyrosol (TY) and hydroxytyrosol (HT), which possess stronger antioxidative activity than OL [41]. As reported, OL and HT reduce hyperglycemia, hyperlipidemia, and insulin resistance (IR) in rats with metabolic syndrome [42,43] and have been proposed as protective against the development of obesity, with the improvement of insulin sensitivity in type 2 diabetes (T2D) [44,45]. Furthermore, it was found that HT reduces the accumulation of fats in the liver and skeletal muscle by a decrease of lipid deposits through inhibition of the sterol regulatory element-binding protein-1c/ fatty acid synthase (SREBP-1c/FAS) pathway [46] and, interestingly, it was shown as more effective than metformin in decreasing glucose and serum lipid levels. OL and HT inhibited the differentiation and adipogenesis of 3 T3-L1 cells in a dose-dependent manner by reducing intracellular triglyceride accumulation, decreasing the expression of PPARγ and C/EBPα and their downstream target genes (CD36, GLUT4), and downregulating the expression of SREBP-1c and its downstream gene (FASN) [46].

### 3.2. Histochemical Analysis of Soleus Muscle in Diabetic Rat after Olive Leaf Polyphenols (OLPs) Treatments

In mammals, skeletal muscles are responsible for providing several functions in metabolism such as energy expenditure, physical strength, and locomotor activity. Decreased muscle mass or atrophy as a result of disturbance of protein degradation in a variety of pathophysiological states, negatively affect the growth, maintenance, and repair of skeletal muscle [47,48]. The lack of reparation of the damaged skeletal muscle as well as the impaired synthesis of muscle creatine kinase and myosins are characteristic for uncontrolled diabetes [48,49,50,51]. Supplementation with antioxidants can protect the muscle tissues and activate different target genes that allow muscle protein synthesis and muscle repair in STZ-induced diabetic rats. Thus, our unpublished results showed that OLP increased glutathione peroxidase (GPx) and superoxide dismutase (SOD) activity in the soleus and tibialis muscles in diabetic rats in a dose-dependent manner.

In pathological or stress conditions, fiber-type switching can occur (e.g., slow- into fast-twitch fibers), such as, for example, in insulin-resistant and diabetic patients. Some phenolic compounds can reprogram of fast-to-slow myofibers’ switch, regulate improving endurance capacity, and alleviate fatigue [52,53]. In addition, GLUT4 is reduced in soleus muscle in diabetic rats [54], thus, we examined the effect of OLP therapy on the recovery of damaged soleus muscle myofibers in diabetic rats. Here, observed morphological changes of the planar muscle confirm that diabetes affected connective tissue density and the presence of nuclei in the endomysium. We also showed that the recovery of damaged fibers was possible using OLP therapy, as demonstrated by an increased number of nuclei. Furthermore, cell renewal and muscle tissue regeneration occurred (see Figure 2D,E), leading to the maintenance of muscle function. We believe that tissue regeneration by OLP occurred after an enhanced initial expression of skeletal proteins such as different myosin isoforms. This finding is supported by Calábria et al. [49], who reported that antioxidant supplementation reduced oxidative stress and regulated myosin protein expression in the diabetic rat brain. This also may be reflected on GLUT4 vesicle trafficking, activation of Rab8A and interaction with myosin, and GLUT4 mobilization to PM [17,55].

### 3.3. Rab8A, Rab13, and Rab14 in Regulation of GLUT4 Translocation in Rat Skeletal Muscle with Diabetes and Following OLP Therapy

Our research is the first to establish a relation between Rabs in a model of GLUT4 trafficking pathways and glucose metabolism in muscle cells in diabetes and the effect of possible therapy on GLUT4 vesicular transport and glucose uptake. Further studies are required to investigate the specific contribution of individual Rab GTPases to glucose uptake and metabolism in muscle cells as well as the relationship between RabGAP–RabGTPase interaction in different types of skeletal muscle fibers.

In this study, we focused on the role of Rab8A, Rab13, and Rab14 in an insulin-stimulated transport of glucose in diabetic rat soleus muscle. Namely, the incorporation of GLUT4 into the plasma membrane is an indicator of the efficacy of intracellular glucose transport into the muscle cell. Although we did not compare the effect of oleuropein, as the major phenolic compound in the olive leaf extract, we assumed that oleuropein from OLP induces GLUT4 translocation to the cell membrane and glucose uptake into the cells. Since the control of muscle glucose uptake allows maintenance of blood glucose homeostasis, we hypothesized that polyphenols can divert glucose into tissues with a greater need or into tissues able to decrease the higher concentration of circulating glucose, as in diabetes. Indeed, as a major insulin-sensitive tissue, skeletal muscle regulates glucose uptake in the whole body and is a target site of glucose level dysregulation, just as it is in the insulin-resistant state [56]. Although the biochemical and histological composition of the muscle is well defined, the functional consequences to adaptations to physiological and pathophysiological conditions and drugs consumption are still not clarified. This relates especially to the intake of natural compounds such as polyphenols.

As observed here, GLUT4 immunofluorescence staining in the rat soleus muscle was seen as dispersed larger bright clusters and smaller punctuate spots. They were found in the perinuclear region and intracellular layers in the healthy control group, where they were less frequent. Opposite to this, in the diabetic control group, in both regions, fewer spots were shown. On the other hand, the formation of small clusters and their uniform incorporation of GLUT4 into the PM was shown in OLP-treated diabetic rats, in the groups TOL2 and TOL3. This dispersive incorporation of GLUT4 could improve glucose uptake into the cells along the cell membrane (see Figure 3A,D–E). Lauritzen et al. [57] observed that after insulin stimulation of rodent muscle, the large GLUT4 clusters did not translocate, they remained stationary and locally depleted, while small clusters could be more mobile and their deposition into the PM more dispersive and uniform. This would mean that the OLP affected the greater GLUT4 expression and motility of the GLUT4 vesicle and its better incorporation into the cell membrane. As reported, in the basal state, at a low rate of continuous insulin supply, only 10% of all GLUT4 structures are mobile, and the rest of GLUT4 is localized near the PM where it can be effective after insulin stimulation of glucose uptake [58]. Additionally, strong evidence suggests that oxidative stress is the major mechanism driving impaired β-cell function and insulin signaling, and phenolic compounds have been shown to exhibit remedial benefits by ameliorating insulin secretion and increase insulin sensitivity. However, whether various polyphenols and phenolic compounds can target specific proteins in an intracellular vesicular glucose transport, which can be involved in the pathogenesis of diabetes, has not been elucidated. Thus, we suggest two main mechanisms by which OLP act on an increase in GLUT4 translocation: β-cell stimulation in the pancreas with ensuing insulin release (regulating the expression of genes associated with insulin secretion and signaling), and insulin-mimetic properties of OLP, which induce the uptake of glucose into muscle and adipose tissue in the absence of insulin or in the presence of insulin resistance. Collectively, our data demonstrate that OLP has a favorable effect on GLUT4 translocation in the skeletal muscle of diabetic rats due to stimulation of GLUT4 mobilization.

Recently, Rabs were proposed as major regulators of cellular division. In particular, they are well-established regulators of membrane transport and have been shown to mediate several membrane transport steps including vesicle formation, molecular motor-dependent vesicle transport, and targeting of transport vesicles and organelles to their correct destinations [3]. It has been shown that mobilization of the vesicle containing GLUT4 to the surface of the muscle cell occurred by the activation of Rab8A and Rab13 [10]. Due to their different cellular functions involved in GLUT4 vesicular transport, we demonstrated the role of specific Rabs in the regulation of glucose transport in diabetic muscle with and without OLP therapy. Here, both Rab8A and Rab13 colocalize with GLUT4 in rat soleus muscle in the TOL3 group, and so, associate with its translocation to the plasma membrane after insulin signaling. We believe that oleuropein promotes GLUT4 translocation, acting as an insulin-mimetic. The interdependence between Rab8A and Rab13 was stated in the String Database (https://string-db.org/cgi/network.pl?taskId=AHeNoXFhAZ4X), where, due to an interaction between them, Rab8A is predicted as a functional partner for Rab13. Based on immunoblot analysis, we found that the expression level of Rab8A, Rab13, and Rab14 significantly correlate with the expression of GLUT4. In addition, the significant correlation was detected between Rab13 and Rab14 (r = 0.8971, *p* = 0.000) in the groups with diabetes (diabetic control group and diabetic OLP-treated groups), while between Rab8A and Rab13, and Rab8A and Rab14, results were not significant. These data suggest that diabetes primarily damages endocytic recycling and regulatory mechanisms for the transport to PM, while the OLP therapy promotes their recovery in a dose-dependent manner. More specifically, Rab13 could represent the major RabGTPase in glucose transport in diabetic soleus muscle upon the OLP intervention due to their function on the tight junction.

Rabs also control motor protein recruitment to their specific target membranes. It is known that skeletal muscle myosin from diabetic rats is more glycosylated and MyoVa reduced compared with healthy control [59]. After activation, Rab8A interacts with the progressive MyoVa and then mobilized the GLUT4 vesicle to the PM [55]. The Rab-regulated step in the GLUT4 trafficking can point to its association with myosin V motors and to its engagement with Rab8A in the muscle cells [17]. Hence, such a finding connects insulin signaling with the Rab8A molecular switch and the motor protein MyoVa to mobilize GLUT4 vesicles toward the PM of the muscle cell [4,60]. We observed here that the very poor colocalization of Rab8A and GLUT4 in the diabetic group resulted in poor glucose incorporation of GLUT4 in the PM, perhaps due to the presence of damaged muscle fibers and reduced level of MyoVa. In addition, Rab8A colocalized with GLUT4 both in the sub-membrane and perinuclear region in the healthy, TOL2, and TOL3 groups, as was visualized by immunofluorescence staining. Probably, cytoskeletal regeneration and reparation occurred stronger by higher-dose OLP. The deficiency of Rab8A in skeletal muscle was also linked with hyperlipidemia and hepatosteatosis in the organism due to impaired muscle lipid uptake and storage [18]. We activated Rabs function by recruiting a distinct set of effectors, such as motor proteins. MICAL-L2 was found as an effector of Rab13, while MyoVa is an effector for Rab8 and Rab14. Given that Rab13 promotes tethering of GLUT4 vesicles near PM after previous interaction with MICAL-L2 [4], the mechanisms proposed for stimulated traffic, tethering, and docking of GLUT4 vesicles unavoidably enroll dynamic actin filaments. Therefore, it could be suggested that OLP therapy induces recovery of the disrupted actin cytoskeleton and improves damaged GLUT4 trafficking in diabetic soleus muscle.

Sun et al. [10] reported that differences in Rab8A and Rab13 time course activation and subcellular localization existed. Namely, Rab8A participates in endosomal transit, while Rab13 regulates peripheral events, i.e., the promotion of GLUT4 vesicles’ fusion with the PM, hence, it is understandable that Rab8A is activated earlier than Rab13. In OLP therapy, we found that activation of Rab8A and Rab13 is linked with the OLP dosage used, such as is presented in Figure 5b for the TOL2 and TOL3 groups. Namely, Rab8A was higher in the TOL2 group, while Rab13 was higher in the TOL3 group. This suggests that the concentration of polyphenols can influence GLUT4 trafficking dynamics. However, additional research is required.

Since diabetes impairs both heavy and light chains of myosin, it seems that it has a negative effect on MyoVa and thus, Rab8A and GLUT4 vesicle movement. On the other hand, the effect of dose-dependent OLP shown in the TOL2 group a significant increase in Rab8A, even more than in the healthy group. There is good evidence that the insulin-stimulated GLUT4 translocation requires Rab8a, Rab13, and Rab14 [10,11] in cultured rat L6 muscle cells. In this study, we demonstrated that OLP stimulated GLUT4 translocation in vivo under the control of Rab8A and Rab13 to regulate GLUT4 vesicle traffic. These novel findings implicate Rab8A and Rab13 as specifically insulin-regulated GLUT4 traffic in rat soleus muscle and demonstrate for the first time the Rab GTPases involved in the intracellular transduction stimulated by polyphenols.

In summary, we investigated the role of polyphenols to stimulate GLUT4 expression and translocation in skeletal muscle in SZT-induced diabetes in rats. Rab8a, Rab13, and Rab14 each contribute in part to OLP-regulated GLUT4 translocation in a dose-dependent manner. Similarities and differences between the actions of different doses of OLP were identified, that support the idea that the concentration of phenolic compounds is crucial for stimulation of distinct pools of GLUT4. Future in vitro and in vivo studies will help explore the downstream effectors of these Rab GTPases and visualize the localization of the responding GLUT4 pools. Finally, these findings will enable to identify potential between the action of natural and conventional therapy and insulin and explore possible avenues to bypass or overcome diabetes.

## 4. Material and Methods

### 4.1. Materials

The chemicals and solvents used were of the HPLC-grade or analytical grade purity. Formic acid, hydrochloric acid (37%), methanol, and acetonitrile (HPLC grade) were purchased from J. T. Baker (Avantor Performance Materials B.V., Deventer, The Netherlands). Citric acid monohydrate (HOC(COOH)(CH_2_COOH)_2_ · H_2_O), sodium citrate tribasic dihydrate (HOC(COONa)(CH_2_COONa)_2_ ·2 H_2_O), streptozotocin, mowiol 4–88, and 1,4-Diazabicyclo[2.2.2]octane (DABCO) were purchased from Sigma–Aldrich (Taufkirchen, Germany). Tris (hydroxymethyl) aminomethane (Tris), sodium chloride (NaCl), monopotassium phosphate (KH_2_PO_4_), and disodium phosphate (Na_2_HPO_4_) were purchased from Prolabo (WVR International, Dublin, Ireland). Triton X-100 was purchased from Merck, KGaA (Darmstadt, Germany). Nonfat dry milk or Blotting-Grade Blocker (Cat.No. 170-6404) was obtained from Bio-Rad (Bio-Rad Laboratories, Inc., Hercules, CA, USA). Oleuropein was purchased from Extrasynthese (Extrasynthese, Genay Cedex, France). Accutrend Plus strips for glucose (Glc), triglycerides (TAG), cholesterol (CHO), and lactate (Lac) were purchased from Roche (Mannheim, Germany).

Mini-PROTEAN^®^ TGX™ Precast Protein Gels (4–15%) and Laemmli Sample Buffer (2X) were purchased from Bio-Rad (Bio-Rad, Hercules, CA, USA). NuPAGE^®^ Sample Reducing Agent (10X) and NuPAGE^®^ Antioxidant were purchased from Novex (Grand Island, NY, USA). PageRuler™ Plus Pre-stained Protein Ladder, 10 to 250 kDa (Cat.No. 26619), Pierce™BCA Protein Assay Kit (Cat.No. 23227), 1-Step™ TMB-Blotting Substrate Solution (Cat.No. 34018), RIPA Lysis and Extraction Buffer (Cat.No. 89900), Halt Protease Inhibitor Cocktail (Part No. 78430), and Halt Phosphatase Inhibitor Cocktail (Part No. 78420) were purchased from Thermo Fisher Scientific (Pierce, Thermo Scientific, Rockford, IL, USA).

Polyclonal antibody to rabbit IgG (H + L chain) secondary antibody (Cat.No. R1364HRP) and rabbit anti-GLUT4 (Cat.No. BP508) were purchased from Acris Antibodies GmbH (Herford, Germany). Primary monoclonal mouse anti-Rab8A IgG1 (sc-81909), mouse anti-Rab13 IgG1 (sc-517224), and mouse anti-Rab14 IgG1 (sc-271401) were donated by Santa Cruz Biotechnology, Inc. (Santa Cruz Biotechnology, Inc., Heidelberg, Germany). The following secondary antibodies were used in this experiment: Donkey anti-Mouse IgG (H + L) Highly Cross-Adsorbed Secondary Antibody, Alexa Fluor 594 (Cat.No. A-21203), and Goat anti-Rabbit IgG (H + L) Cross-Adsorbed Secondary Antibody, Alexa Fluor 488 (Cat.No. A-11008), were purchased from Invitrogen (Eugene, OR, USA). Normal goat serum was purchased from Dako (Agilent Pathology Solutions, Santa Clara, CA, USA).

DAPI (4’,6-Diamidino-2-Phenylindole, Dihydrochloride) (Cat.No. D1306) was purchased from Invitrogen (Eugene, OR, USA). Polyvinylidene difluoride (PVDF) membrane and blocking reagent were obtained from Roche Diagnostics GmbH (Mannheim, Germany). Adhesive microscope slides were purchased from Biognost (Zagreb, Croatia).

### 4.2. Phenolic Extraction and Analysis

#### 4.2.1. Preparation of the Olea Europaea Leaf Extract (OLE)

Olive leaf extract (OLE) was prepared from samples of olive-tree leaves picked from the Busa variety grown in Vodnjan (in southwest Istria, Croatia, 44°57′40″ N–13°51′10″ E) on 20 November 2014 according to the method of Giacometti et al. [61]. The dry residue was weighed, dissolved in sterile saline, and kept at −20 °C until its use as therapy.

In addition, the sample of OLE was filtered through a 0.2 µm nylon syringe filter and a concentration of oleuropein was determined using ultra-high-pressure liquid chromatography with a diode array detector (UHPLC-DAD).

#### 4.2.2. UHPLC-DAD Analysis of Oleuropein in the OLE

The Agilent 1290 Infinity LC system (Agilent Technologies, Palo Alto, CA, USA) equipped with ChemStation software and a diode-array detection (DAD) system were used for olive leaf extract analysis according to the method of Giacometti et al. [61]. The identification of oleuropein in OLE was carried out at 280 nm and quantification was performed using a calibration curve of oleuropein (y = 0.6845x − 3.8638, R^2^ = 0.9994). The result was expressed as the mean values of two independent experiments (n = 2).

### 4.3. Experimental Protocols

#### 4.3.1. Animals Experimental Design and Treatments

All experimental procedures were conducted in accordance with the European Directive 86/609/EEC, the Recommendation 2007/526/65/EC approved by the Ethics Committee of the Department of Biotechnology, University of Rijeka (Kl.: 644-01/16-01/03-01, Ur.br.: 2170-57-005-01-16-5 from 14 September 2016). All efforts were made to minimize animal suffering and to reduce the number of animals used, in accordance with the commonly accepted ‘3Rs’ (Replacement, Reduction and Refinement). Animals were obtained from the Institute for Medical Research and Occupational Health (Zagreb, Croatia). After the acclimatization period, rats were caged in groups under controlled conditions (22 ± 1 °C, 50% ± 5% humidity and 12 h light–dark cycles), fed a standard rodent diet (pellet, type 4RF21 GLP, Mucedola, Italy) and water ad libitum. In addition, before and after each treatment throughout the study, rats were weighed.

Healthy male Wistar rats weighing 200–220 g at 9 weeks old were randomly divided into five groups with 6 animals each, as follows: a non-diabetic healthy group (Control), a diabetic group (DM group), and three diabetic groups treated with OLE in a dose-dependent manner, TOL1, TOL2, and TOL3 (512, 768, and 1024 mg/kg, respectively) (see Appendix A Figure A1). Rats were fasted for 8 h before the experiment and had free access to water. Diabetes was induced in rats intraperitoneally (*i.p.*) by a single administration of streptozotocin (SZT, 60 mg/kg of BW) dissolved in a citrate buffer (0.01 M, pH 4.5). The citrate buffer alone was injected into the Control animals. Rats were considered diabetic once their fasting blood glucose (FBG) levels had reached or exceeded a concentration of 225 mg/dL on five consecutive days. The intraperitoneal (*i.p.*) administration of OLE started on the 8th day after SZT injection in the dose-dependent groups, TOL1, TOL2, and TOL3, with 512, 768, and 1024 mg/kg daily for ten days. The concentration of oleuropein in the injected OLE was calculated based on UHPLC-DAD analysis as follows: in OLE by 1024, 768, and 512 mg/kg, were 44.5, 33, and 20.3 mg/kg of oleuropein, respectively.

The blood glucose was monitored at set time intervals by measuring blood glucose concentration in blood samples withdrawn from a tail vein using the Bionime GM550 Blood Glucose Monitoring System (Bionime Corp., Taichung City, Taiwan). Diabetic animals were narcotized and sacrificed on the 8th day after SZT injection.

Four hours after the last OLE dose was administered, rats were euthanized by an overdose of the mixture of ketamine/xylazine solution (10:1). Blood was collected from the orbital sinus. Thereafter, the soleus muscle was dissected, divided into samples, and immediately frozen in liquid nitrogen and stored at −80 °C.

#### 4.3.2. Glucose Tolerance Test (GTT)

Glucose tolerance tests were performed separately on 9-week-old male rats after 8 h fasting before being the administration of glucose and OLP extracts. Normal rats were divided into four groups (each group 5 rats) for intraperitoneal (IPGTT) administration by glucose (2 g/kg BW) and three doses of OLP extracts (512, 768, and 1024 mg/kg BW). After two days of the SZT administration, diabetic rats were screened for diabetes with blood glucose levels. The blood glucose level of 238.4 ± 23.6 mg/dL was taken for the study. Diabetic rats were also divided into four groups (each group 5 rats), administrated glucose (2 mg/g body weight), and three doses of OLP extracts (512, 768, and 1024 mg/kg BW). GTT was carried out 2 h after the initial OLP administration in diabetic rats. Blood samples were collected from the tail vein at 0, 30, 60, and 120 min, and blood glucose was determined using the Bionime GM550 glucometer.

#### 4.3.3. Blood Biochemistry

Immediately after blood samples were taken, the concentration of glucose (Glc), triglycerides (TAG), and cholesterol (CHO) were measured using the corresponding assay strips (Accutrend Plus, Roche, Mannheim, Germany).

#### 4.3.4. Tissue Muscle Homogenization

Muscle samples were cut with scissors, lysed with the cryo-pulverization method using liquid nitrogen, and then suspended in a radioimmunoprecipitation assay (RIPA) buffer that contained protease and phosphatase inhibitors. Then, homogenates were centrifuged in an Eppendorf 5427R centrifuge (Eppendorf, Hamburg, Germany) for ten min at 5000 rpm and 4 °C. Supernatants were aliquoted and stored at −80 °C until analysis.

Protein concentrations in supernatants of muscle homogenate were determined according to the manufacturer’s procedure using a BCA protein assay kit.

#### 4.3.5. H&E and Immunofluorescence Staining

Histopathological assessment of muscle alteration structure was performed by studying hematoxylins and eosin (H&E)-stained slides. Several hematoxylin and eosin-stained sections (7 μm) at the soleus muscle were prepared to characterize skeletal muscle pathology. To evaluate the histopathological damage, images of stained sections were done with magnification at 40× and/or 10× using a digital camera (Olympus DP70) attached to a light microscope (Olympus BX51 microscope). Three different researchers examined all slides in a blinded manner.

Immunofluorescence staining was performed on frozen tissue sections as described by Giacometti and Grubić-Kezele [62]. Briefly, frozen soleus muscle biopsy samples were cut transversally using a cryostat to a thickness of 5 µm with a Leica CM1850 UV Cryostat (Leica Biosystems, Nussloch, Germany) onto uncoated glass microscope slides (VWR international, Leicester, UK). Tissue sections were dried for 30 min at room temperature and then fixed with pre-cooled acetone (−20 °C) for 10 min. After immersion of tissue sections, acetone was evaporated from slides for 20 min at room temperature and then rinsed in phosphate-buffered saline (PBS) solution at a neutral pH. Nonspecific binding was blocked by one-hour incubation with 1% BSA in a PBS containing 0.001% NaN_3_ at room temperature. The following primary antibodies were used: rabbit anti-GLUT4 IgG (dilution, 1:100), mouse anti-Rab8A IgG1 (dilution, 1:50), mouse anti-Rab13 (dilution, 1:50), and mouse anti-Rab14 (dilution, 1:50). Primary antibodies were diluted in the blocking solution and incubated with tissue sections overnight at 4 °C in a humid environment. Alexa Fluor donkey anti-mouse IgG 594 nm (dilution, 1:500) and Alexa Fluor goat anti-rabbit IgG 488 nm (dilution, 1:300) were used as secondary antibodies to visualize immunocomplexes. Secondary antibodies were diluted in blocking solution and incubated with tissue sections in the dark at room temperature for 1 h in a humid environment. Nuclei were visualized with 4′, 6-diamidino-2-phenylindole, dihydrochloride (DAPI). Images were taken with the Olympus imaging system BX51 equipped with a DP71CCD camera (Olympus, Tokyo, Japan) and processed with CellF imaging software. Double immunolabeling was performed in soleus muscle cells for GLUT4 and Rab8A, GLUT4 and Rab13, and GLUT4 and Rab14. Quantitative measurements for the extent of colocalization of two signals in double immunolabeling were analyzed using the JaCoP plug-in for ImageJ to calculate the Pearson’s coefficient.

#### 4.3.6. SDS-PAGE and Western Blot

50 µg of protein was subjected to SDS-PAGE and transferred to a PVDF membrane using a semi-dry protocol after previous protein determination by the BCA method. Electrophoretic separation was performed using precast 4–15% TGX (Tris-Glycine eXtended) gels in the Mini-PROTEAN Tetra Vertical Electrophoresis Cell (Bio-Rad, Hercules, CA, USA) according to the manufacturer’s procedure.

The transfer run was at 100 mA for 1 h in an SD10 semi-dry blotter (Cleaver Scientific Ltd., Rugby, Warwickshire, UK). The membranes were blocked in TBST (tris-buffered saline and polysorbate 20) with a 5% *w/v* nonfat dry milk, incubated with primary antibodies GLUT4 (at 4 °C, dilution 1:1000), Rab8A (at 4 °C, dilution 1:500), and Rab13 (at 4 °C, dilution 1:500) overnight with agitation. After that, membranes were washed three times for 5 min with TBST with agitation and incubated for 2 h at room temperature with the appropriate horseradish peroxidase (HRP)-secondary antibody (dilution 1:1000, with agitation). Next, they were washed with TBST again, three times, for 5 min at room temperature. The membranes were stained by chromogen using a 1-Step TMB-Blotting Substrate Solution at room temperature, while protein bands’ intensity were visualized with the VersaDoc Imaging System and the bands were analyzed using the Gel Analyzer tool from ImageJ. Data were expressed as normalized expression levels of the DM, based on the band intensity of the protein of interest.

#### 4.3.7. Statistical Analysis

The data were evaluated with Statistica (data analysis software system), version 13 (TIBCO Software Inc., 2017, Palo Alto, CA, USA). The distribution of data was tested for normality using the Kolmogorov–Smirnov test. Differences between groups were assessed with either one-way analysis of variance (ANOVA) followed by the post hoc Scheffé test or the nonparametric Kruskal–Wallis ANOVA by Ranks and Mann–Whitney U test. Pearson correlation (r) was used for determining the association between GLUT4 and Rab8A, GLUT4 and Rab13, and GLUT4 and Rab14 skeletal muscle protein expressions within immunofluorescence images. Statistical significance was assumed, given *p* < 0.050, and the data are reported based on the mean (± SD).

## 5. Conclusions and Future Research

The present study demonstrated that olive leaf polyphenols could improve glucose translocation in skeletal muscle and influence glucose uptake as the treatment for streptozotocin (SZT)-induced diabetes. The use of OLE decreased blood glucose and triglyceride levels in a dose-dependent manner. Moreover, the OLE increased the expression levels of Rab8A, Rab13, and Rab14 proteins.

This is the first investigation with clear evidence that the OLE, as a potential insulin stimulator, can be used for the treatment of diabetes via the improvement of intracellular GLUT4 translocation in the skeletal muscle by the activation of Rab8A and Rab13 proteins.

It is important to note that this approach in the investigation of polyphenolic action on the intracellular GLUT4 translocation mechanism does not yet exist. The current study has limitations with respect to the antidiabetic activities of the OLE towards the rat model induced by SZT such as the hormonal state of animals in the studied state, including measuring the insulin blood level.

Furthermore, since GLUT4 trafficking occurs very rapidly, new techniques, both spatially and temporally, are greatly needed to determine the dynamics of GLUT4 translocation and their individual role in insulin-stimulated GLUT4 vesicle trafficking. Therefore, future investigations are appreciated to more thoroughly reveal the effect of the OLE on different steps involved in GLUT4 trafficking and in our further research, are directed to the Rab motor and SNARE (Soluble N Ethylmaleimide Sensitive Factor Attachment Protein Receptor) proteins that are potentially involved in the regulation of vesicle trafficking. This will complement the application of knowledge for the use of phytotherapy in the management of diabetes mellitus and other diseases with deranged glucose homeostasis.

## Figures and Tables

**Table 1:** Body weight and blood biochemistry.

	Group				
	Control	DM	TOL1	TOL2	TOL3
Weight, g	298.8 ± 21.38	241.6 ± 37.45 #	220.0 ± 20.00	240.67 ± 31.88	235.3 ± 4.51
Glucose, mg/dL	116 ± 22.3	309 ± 40.7 #	261 ± 8.3 *	228.2 ± 36.9 *	257 ± 27.9 *
Cholesterol, mg/dL	165 ± 6.2	166 ± 4.3	163 ± 5.1	160 ± 1.1	164 ± 6.9
Triglycerides, mg/dL	104 ± 2.1	193 ± 114.1 #	113 ± 7.6 *	119.5 ± 4.7	100 ± 15.5 *

TOL1-diabetic group treated with 512 mg/kg olive leaf extract (OLE), TOL2-diabetic group treated with 768 mg/kg olive leaf extract (OLE), and TOL3-diabetic group treated with 1024 mg/kg olive leaf extract (OLE). # significantly different from the Control; * significant difference between the DM group and treated groups (TOL1, TOL2, TOL3). The difference was significant at *p* < 0.050.

## Abbreviations

ACC	Acetyl-CoA carboxylase
AMPK	5’ adenosine monophosphate-activated protein kinase
AS160 (or TBC1D4)	160 kDa substrate of the Akt Ser/Thr kinase
C/EBPα	CCAAT/enhancer-binding protein alpha is a transcription factor
CPT-1	Carnitine O-palmitoyltransferase 1
DM	Diabetes mellitus
ERK1/2	Extracellular signal-regulated kinases 1 and 2
FASN	Fatty acid synthase gene
GGT	Glucose tolerance test
GLUT1	Glucose transporter 1
GLUT4	Glucose transporter 4
GSV	Intracellular storage vesicles
GTPases	Guanosine triphosphate (GTP) ases
HbA1c	Glycated hemoglobin A 1c
HDL-C	High-density lipoprotein cholesterol
IL-10	Interleukin-10
IL-6	Interleukin-6
IR	Insulin receptor
iNOS	Nitric oxide synthase, inducible
IRS-1	Insulin receptor substrate 1
JAK/STAT	Janus kinases/signal transducer and activator of transcription proteins
LDL-C	Low-density lipoprotein cholesterol
MAPK3/1	Mitogen-Activated Protein Kinase 3 and 1
MCAD	Medium-chain specific acyl-CoA dehydrogenase
MCP-1	Monocyte chemoattractant protein 1
MyoVa	Myosin V heavy-chain gene (a class of actin-based motor proteins)
NEFA	Non-esterified fatty acids
NF-κB	Nuclear factor NF-kappa-B
OLE	Olive leaf extract
p-ACC	Phospho-Acetyl-CoA carboxylase
p-Akt	Phosphorylated version of AKT
p-AMPK	Phospho-AMPK
PARP	Poly (ADP-ribose) polymerase
PEPCK	Phosphoenolpyruvate carboxykinase
p-ERα	Phosphorylated estrogen receptor alpha
PGC-1α	Peroxisome proliferator-activated receptor gamma co-activator 1 alpha
p-GSK-3β	Phosphorylated glycogen synthase kinase-3 beta
p-IR	Phosphorylated insulin receptor
PI3K/AKT	Phosphatidylinositol 3-kinase (PI3K)/protein kinase B (AKT) signaling pathway
p-JNK	Phospho-JNK
PKC λ/ξ	Protein kinase C λ/ξ
PLC-PKC	Phospholipase C-protein kinase C
PM	Plasma membrane
p-mTOR	Phosphorylated mammalian target of rapamycin (mTOR)
p-p38 MAPK	Phospho-p38 MAPK
PPARγ	Peroxisome proliferator-activated receptor gamma
PPARα	Peroxisome proliferator-activated receptor alpha
p-PKC	Phosphorylated protein kinase C
Rab13	Ras-related protein Rab-13
Rab14	Ras-related protein Rab-14
Rab8A	Ras-related protein Rab-8A
Rac 1	Ras-related C3 botulinum toxin substrate 1
SREBP-1c	Sterol regulatory element-binding protein-1c
SZT	Streptozotocin
TNFα	Tumor necrosis factor alpha
TOL1	Treatment with 512 mg/kg OLE
TOL2	Treatment with 768 mg/kg OLE
TOL3	Treatment with 1024 mg/kg OLE

## Appendix A

**Table A1:** Summary of dietary stimulators of GLUT4 expression, translocation, and physiological adaptions of skeletal muscle in in vitro and in vivo experimental models.

Compound	Model	Protein Expression	Glucose Uptake (GU)/ Blood Glucose (BG)/ Insulin Sensitivity (IS)	Additional Findings	Treatment	Reference
Chlorogenic acid	L6 myotubes	GLUT4	GU	↑ PPARγ protein	25 μM for 5 h: cytotoxicity > (50 μM)	[63]
Cinnamon and extracts	C2C12 myotubes	p-AMPK	GU		100 or 1000 μg/mL for 3 h	[64]
	C2C12 myotubes				30 μg/mL for 4 h	[65]
	Wistar rats (WR) (gastrocnemius muscle)	GLUT4		↑ NEFA (serum), ↓ serum creatine ↑ GLUT4	30 mg/kg/day for 22 days	[66]
	C57BL/6J mice		BG	↓ NEFA (serum), ↓ LDL-C, ↓ insulin	Overnight fast. 400 mg/kg/day for 21 days	[67]
	db/db mice		BG	↓ NEFA (serum) ↓ fasting blood glucose level	6 h fast. 400 mg/kg/day for 14 days	
	C57BL/6J mice		BG	↑ IRS-1, IR protein	Overnight fast. 150 mg/kg/day for 14 days	[68]
	C57BLKS/J db/db mice		BG	↑ HDL-C levels (serum) ↓ p-Akt, Upregulated mRNA GLUT4 ↑ p-Akt	20 mg/kg/day (p.o.) for 4 weeks	[69]
Curcumin	C2C12 myotubes	p-Akt, p-AMPK		↑ p-ACC protein ↑ GU ↑ GLUT4 translocation ↑ p-AMPK ↑ p-ACC ↑ p-Akt (insulin-induced)	40 μM for 24 h: cytotoxicity > 40 μM 40 µM for 1 h	[70]
	WR (soleus muscle)	p-AMPK	BG		1 μM for 30 min or 60 mg/kg	[71]
	C2C12 cells			↑ GU ↓ p-IRS-1 ↓ p-ACC ↑ p-Akt ↑ p-ERK1/2 ↑ p-p38 MAPK	20 µM for 2 h	[72]
	C2 murine myoblasts			↑ Apoptosis ↓ Cell viability ↑ PARP fragmentation ↑ p-JNK	50 µM for 24 h	[73]
	L6myc skeletal muscle cells			↑ GLUT4 translocation ↑ p-Akt ↑ p-GSK-3β ↓ TNF-α, IL-6 and MCP-1 levels ↑ IL-10 levels	25 µM for 16 h	[74]
ECGC	L6 myotubes	p-Akt, p-AMPK	GU		40 μM for 3 h	[75]
	SD rats (soleus muscle)				12 h fast. 75 mg/kg for 1 h, or 100 nM for 15 min	[29]
	C57BL/6 mice (soleus muscle)				12 h fast. 75 mg/kg for 1 h, or 100 nM for 15 min	
	L6 myotubes		GU		100 nM for 15 min	
	C2C12 myotubes	p-Akt, p-AMPK		↑ p-p38 MAPK, ↑ p-ACC	20 μM up to 72 h: treatment not cytotoxic up to 48 h	[72]
	L6 myotube	GLUT4		↑ PI3K, ↓ PKC λ/ξ ↑ GLUT4 RNA, ↑ Rac1	1 nM	[76]
	ICR mice	GLUT4, PI3K, p-AMPK		↑ GLUT4, glycogen accumulation in skeletal muscle	Oral administration postprandial hyperglycemia	
	C2C12 myotubes (ßGlud1−/−)	p-AMPK		↑ p-AMPK, ↑ GU, ↑ IS	20 µM EGCG for 10 min	[77]
Ellagic acid	3T3-L1 adipocytes and C2C12 myotubes	GLUT4, p-AMPK	GU	↑ GLUT4 ↑ p-AMPK ↑ p-ERK1/2 ↑ p-PKC ζ/λ	50 μg/mL for 1 h 1, 10, 100, 500 nM	[33]
Ferulic acid	L6 myotubes	GLUT4	GU	↑ PI3K protein	5 μM for 5 h: cytotoxicity > (50 μM)	[63]
Gingerol	L6 myotubes		GU		40 μg/mL for 48 h: treatment not cytotoxic	[78]
Naringenin	L6 myotubes	p-AMPK	GU		150 μM for 2 h	[79]
Quercetin	ob/ob mice (gastrocnemius muscle)	GLUT4	BG, IS	↑ GLUT4 RNA, ↓ TNF-α, ↓ iNOS RNA, ↓ NF κB activation	30 mg/kg alternating days for 10 weeks	[80]
	L6 myotubes	p-Akt	GU		200 μM for 48 h	
	Kunming mice (gastrocnemius muscle)	GLUT4, p-AMPK	BG	↑ p-ACC, ↓ blood triacylglycerol, ↓ total cholesterol,	12-h fast. 5, 10, 20 mg/kg/day for 13 weeks	[81]
	C2C12 myotubes	GLUT4, p-AMPK	GU	↑ PPARα, ACC, MCAD, CPT-1, GLUT4, PGC-1α RNA, p-ACC protein	10 μM for 24 h: treatment not cytotoxic	
	C2C12 myotubes		GU	↑ p-ACC protein	100 μM for 18 h	[81]
	C2C12 myotubes	p-AMPK	GU	enhanced glucose uptake by 38–59% stimulated uptake by 37%, stimulated the AMPK pathway (25–100 mM) inhibited ATP synthase (in mitochondria) by 34 and 79%	quercetin-3-O-glycosides (50 mM; 18 h treatment) in the absence of insulin quercetin aglycone and quercetin glycosides quercetin aglycone by 25 and 100 mM	[82]
	L6 myotubes	CaMKKβ/AMPK, IRS1/PI3K/Akt JAK/STAT	GU	↑ GU (0.1 nM and 1 nM quercetin or 1 nM isorhamnetin) ↑ JAK/STAT (1 nM and 10 nM isorhamnetin) ↑ p-AMPK (quercetin) ↑ JAK2/STAT (isorhamnetin) ↑ IRS-1 (at 10 nM)		[34]
	ICR mice	GLUT4	GU	quercetin aglycone form were 4.95 and 6.80 nM (plasma concentration)	10, 100 and 1000 mg/kg body weight	
Resveratrol	L6 myotubes	p-AMPK	GU		100 μM for 4 h	[83]
	db/db mice		BG	↑ Glucose tolerance	5 mg/mL/100g body weight for 3 weeks †	
	SD rats (soleus muscle)		GU, BR		Overnight fast. 10 mg/kg/day for 16 weeks	[84]
	C2C12 myotubes		GU		10 μM for 24 h	
	L6 myotubes	p-AMPK	GU		100 μM for 2 h: cell morphology unaltered up to 125 μM	[85]
	SD rats (soleus muscle)		GU	↑ p-ERα, ↑ p-IR, ↓ serum cholesterol, ↓ triglycerides, ↓ uric acid	1 mg/kg/day for 15 days or 15 weeks	[86]
	C2C12 myotubes	p-Akt	GU	↑ p-ERα, ↑ p-p38 MAPK, ↑ p-ERK, ↑ p-IR	0.1 μM for 14 h: treatment not cytotoxic	
	WR (soleus muscle)	GLUT4, p-Akt	BG	↑ PEPCK	Overnight fast. 0.05–10 mg/kg/day for 7 days	[87]
	C2C12 myotubes		GU		30 μM for 30min	
	C2C12 myocytes	p-AMPK		↑ PGC-1α RNA	50 μM for 24 h: cytotoxicity > (50 μM)	[88]
	SIRT1 knockout mice		BG, IS	↓Mitochondrial content and respiration	100 mg/kg day for 9 weeks	[89]
Green tea	Wistar rats (WR)	GLUT4		↓ Triacylglycerols (plasma) ↓ NEFA, ↓ HbA1c, ↑ GLUT4	50 mg/kg body weight for 12 days	[90]
	KK-Ay mice	GLUT4	IS	↓ Triacylglycerols (plasma)	4 weeks	
Procyanidins (dimer to tetramer) from black soybean seeds	ICR mice (soleus muscle)	GLUT4	GU	↑ GLUT4 ↑ p-IRS-1 ↑ p-AMPK ↑ plasma insulin level ↑ plasma adiponectin ↓ plasma glucose	EC and C3G in water at 10 μg/kg body weight	[91]
Procyanidins from cocoa liquor (CLPr)	L6 myoblasts	GLUT4	GU	↑ GLUT4 (7 days) GLUT1 unchanged	250 mg/mL CLPr in DMSO	[92]
	ICR mice (soleus muscle)	GLUT4	GU	↑ GLUT4 (7 days) GLUT1 unchanged	1, 5, 10 μg/mL single oral administration	
High-molecular-weight cocoa procyanidins	human primary skeletal muscle cells	GLUT4, PI3K/AKT, p-AMPK	GU	↑ glycogen synthesis (cocoa extract), ↑ glycogen synthase (GS) (monomers 30% at 10 μM, oligomers 62% at 10 μM, polymers 16% at 10 μM and 32% at 25 μM) ↑ GU (all doses) ↓ PI3K/AKT (CE, 25 μM; oligomer, 25 μM; polymer 10 and 25 μM)	10 and 25 μM	[93]
Sinapic acid	SZT-diabetic rats (soleus muscle)	GLUT4	GU	↑ reduced glucose infusion rate (GIR)	5 mg/kg, 10 mg/kg, and 25 mg/kg	[94]
	L6 cells		GU	↓ PLC-PKC signals ↑ glucose uptake		
Gallic acid	3T3-L1 cells	GLUT4	GU	↑ GLUT4 ↑ PKCζ/λ	1 µM, 10 µM, 20 µM	[95]
	HFD SZT-diabetic rats (adipose tissue)	GLUT4	GU	↑ PPARγ ↑ GLUT4 ↑ PI3K/p-Akt ↑ GU	20 mg/kg	[96]
Black tea polyphenols (theaflavin)	L6 myotubes	GLUT4, p-AMPK	GU	↑ p-IRS1 ↑ p-AMPK/AMPK ↑ p-GSK-3β ↑ PI3K	0.1, 1.0, 10 BTP µg/mL	[32]
Rosemary extract	L6 myotubes	p-AMPK, GLUT4	GU	↑ GU (5 µg/mL) ↑ p-AMPK ↑ p-ACC protein ↑ p-PKC	5 µM RA for 4 h (maximum)	[97]
Carnosol	L6 myotubes GLUT4myc	p-AMPK, GLUT4, p-Akt	GU	↑ p-Akt ↑ p-mTOR ↑ p-ACC protein ↑ GLUT4	25 µM carnosol (4 h)	[31]
